# Some brief comments on animal rights

**DOI:** 10.1093/af/vfz050

**Published:** 2020-01-10

**Authors:** Gary L Francione

**Affiliations:** Rutgers University School of Law, Camden/Newark, New Jersey School of History and Heritage, Philosophy Department, University of Lincoln (U.K.)

**Keywords:** animal rights, animal welfare, meat, property, slavery

**Implications**

- It is absurd that some animal rights campaigners maintain that we should allow animals the same legal rights enjoyed by humans.
- A sensible and coherent theory of animal rights should focus on just *one* right for animals—the right not to be treated as the property of humans.
- Recognizing animal rights really means accepting that we have a duty not to treat sentient nonhumans as resources.
- There is no necessity for 99% of our animal uses.

Do animals have moral rights? What kind of legal status should we afford them? The debate on these issues has become very confused. Some animal rights campaigners maintain that we should allow animals the same legal rights enjoyed by humans (Figure 1). That is, of course, absurd. There are many human rights that simply have no application to nonhumans.

**Figure 1.**
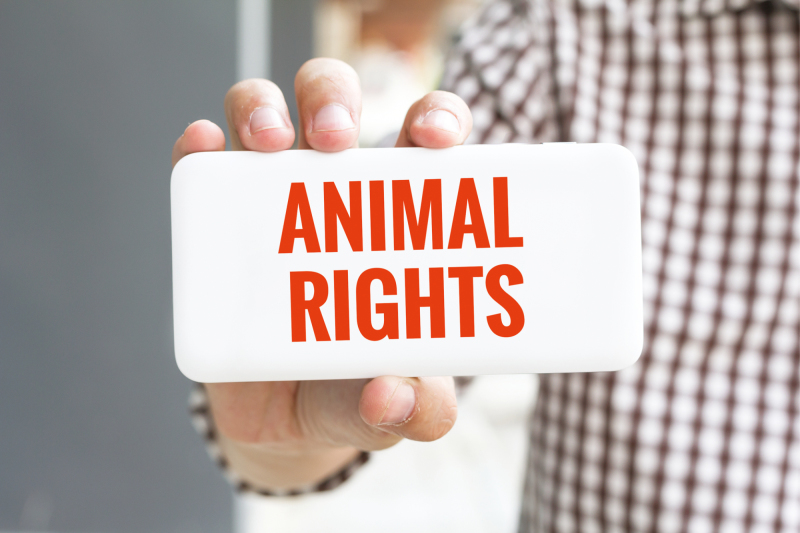
Do animals have moral rights?

In the work that I have done on animal rights (Francione, 2000, 2008), I propose that a sensible and coherent theory of animal rights should focus on just *one* right for animals—the right not to be treated as the property of humans.

Let me explain why this makes sense. At present, animals are property; they are commodities that we own in the same way that we own automobiles or furniture. Like these inanimate forms of property, animals have only the value that we choose to give them. Any interest an animal has represents an economic cost that we as owners can choose to ignore (Figure 2).

**Figure 2.**
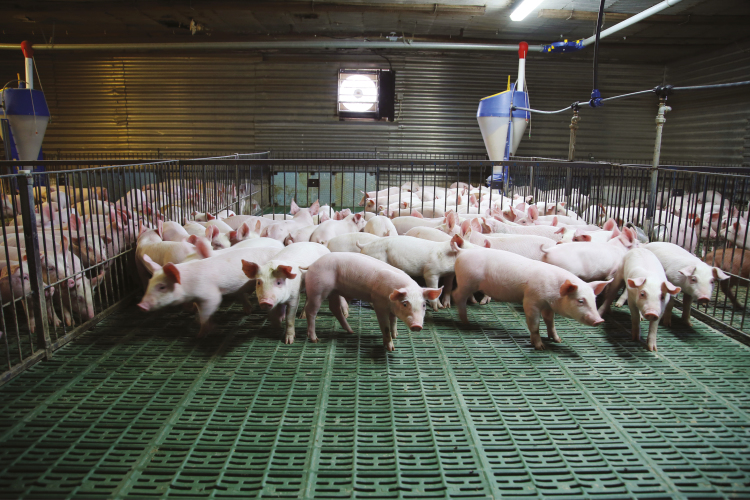
Young domestic piglets growing in a modern swine production farm.

Animal property is, of course, different from the other things that we own in that animals, unlike cars, computers, machinery, or other commodities, are sentient and have interests (Figure 3). All sentient beings have interests in not suffering pain or other deprivations, and in satisfying those interests that are peculiar to their species. It costs money to protect animal interests. As a general matter, we spend money to protect animal interests only when it is justified as an economic matter—only when we derive an economic benefit from doing so (Francione, 1995). The result is that animal welfare standards do little more than ensure that animals are exploited in an economically efficient way.

**Figure 3.**
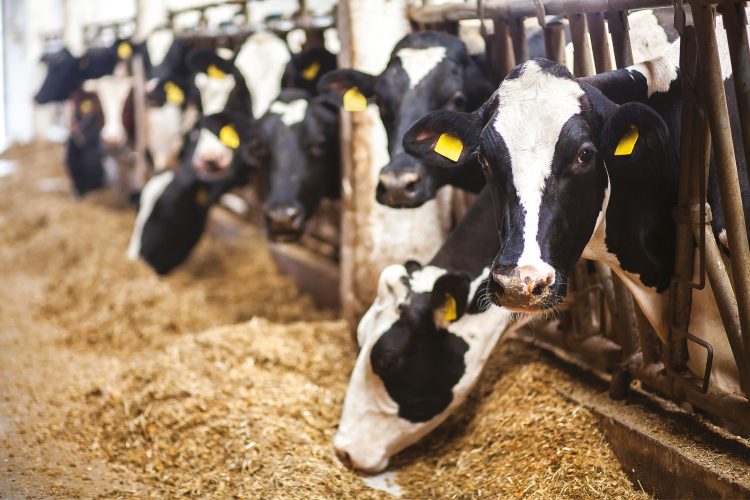
Dairy cattle eating a standard high-energy ration.

For example, the Humane Slaughter Act in the United States, enacted originally in 1958, requires that larger animals slaughtered for food be stunned and not be conscious when they are shackled, hoisted, and taken to the killing floor. This law protects the interests that animals have at the moment of slaughter but does so only because it is economically beneficial for producers and consumers. The “[f]indings and declarations of policy” of the Act state: “The Congress finds that the use of humane methods in the slaughter of livestock prevents needless suffering; results in safer and better working conditions for persons engaged in the slaughtering industry; brings about improvement of products and economies in slaughtering operations; and produces other benefits for producers, processors, and consumers which tend to expedite an orderly flow of livestock and livestock products in interstate and foreign commerce.” 7 U.S.C. § 1901 (2000).

Large animals who are conscious and hanging upside down and thrashing as they are slaughtered will cause injuries to slaughterhouse workers and will incur expensive carcass damage. Therefore, stunning large animals makes good economic sense. Of course, these animals have many other interests throughout their lives, including an interest in avoiding pain and suffering at times other than at the moment of slaughter, and these other interests are not protected because it is not economically efficient to do so. Moreover, the Humane Slaughter Act has not been interpreted to apply to smaller animals, including birds, who account for about 95% of the animals slaughtered for food in the United States. The reason for this exclusion is that given the number of birds slaughtered, and their relatively smaller size and lesser value, it has not been considered economically efficient to protect their interests in the same way as the interests of larger animals. Many welfarist campaigns promote reforms explicitly based on economic efficiency and increased productivity. For example, the campaign to replace conventional chicken slaughtering with asphyxiation is promoted on the ground that the latter will increase production efficiency for industry (Francione and Garner, 2010).

There are laws—anticruelty laws—that require that we treat animals “humanely” and that we do not inflict “unnecessary” suffering on them. In many cases, these laws carry criminal sanctions (albeit minor ones) for violation. For the most part, only laws that reflect widely accepted moral norms are contained in criminal codes. Therefore, it can be said that, on one level, we take the idea of the “humane” treatment of animals seriously. These laws, however, do not actually prohibit uses of animals that are unnecessary; instead, at most, they prohibit treatment that is customarily avoided by institutional animal users because that treatment is economically inefficient. *So we do not ask whether animal use is necessary; we assume that animal use is acceptable and simply ask about whether it is necessary to treat animals in a particular way in order to use them as property.* These laws require that we balance the interests of humans and animals in order to ensure that animals are treated “humanely.” It is, however, a fallacy to suppose that we can balance human interests, which are protected by claims of right in general and of a right to own property in particular, against the interests of animals which, as property, exist only as a means to the ends of humans. The animal in question is always a “pet” or a “laboratory animal” or a “game animal” or a “food animal” or a “circus animal” or some other form of animal property that exists solely for our use. We prohibit animal suffering only when it has no economic benefit. The balance is unbalanced from the outset.

There are parallels here with the institution of human slavery. While we tolerate varying degrees of human exploitation (wrongly, in my view), we no longer regard it as legitimate to treat anyone, irrespective of their particular characteristics, as the property of others. In a world deeply divided on many moral issues, one of the few norms steadfastly endorsed by the international community is the prohibition of human slavery. Some forms of slavery are worse than others, yet we prohibit all of them—however “humane”—because they all more or less allow the fundamental interests of slaves to be ignored if it provides a benefit to slave owners. We recognize all humans as having a basic right not to be treated as the property of others. This is not to say that slavery no longer exists; it does. But no one defends it.

Is there a morally sound reason not to extend this single right—the right not to be treated as property—to animals? Or to ask the question another way, why do we deem it acceptable to eat animals, hunt them, confine and display them in circuses and zoos, use them in experiments or rodeos, or otherwise treat them in ways in which we would never think it appropriate to treat any human irrespective of how “humane” we were being?

The usual response that animals lack some special characteristic that is possessed solely by humans not only flies in the face of the theory of evolution but is completely irrelevant to whether it is morally permissible to treat nonhumans as commodities—just as differences among humans would not serve to justify treating some as slaves. The differences between a normally functioning human and a severely disabled human may be relevant insofar as they justify differential treatment. We may provide access to certain benefits, such as a university education, to one that we would deny to the other. But we would not conclude that it is appropriate to use the disabled human as a chattel slave or as forced organ donor.

Also of no use is the response that it is acceptable for humans to exploit nonhumans because it is “traditional” or “natural” to do so. This merely states a conclusion and does not constitute an argument. There is very little that we now see as morally objectionable that was not once considered as “traditional” or “natural.”

The bottom line is that we cannot justify human domination of nonhumans. Our “conflicts” with animals are mostly of our own doing. We bring billions of sentient animals into the world in order to kill them for reasons that are often trivial. We then seek to understand the nature of our moral obligations to these animals. But by bringing these animals into existence for reasons that we would never consider appropriate for humans, we have already decided that animals are outside the scope of our moral community altogether. Accepting that animals have this one right does not entail letting cows, chickens, pigs, and dogs run free in the streets (Figure 4). We have brought these animals into existence and they depend on us for their survival. We should care for those currently in existence, but we should stop causing more to come into being to serve as our resources.

**Figure 4.**
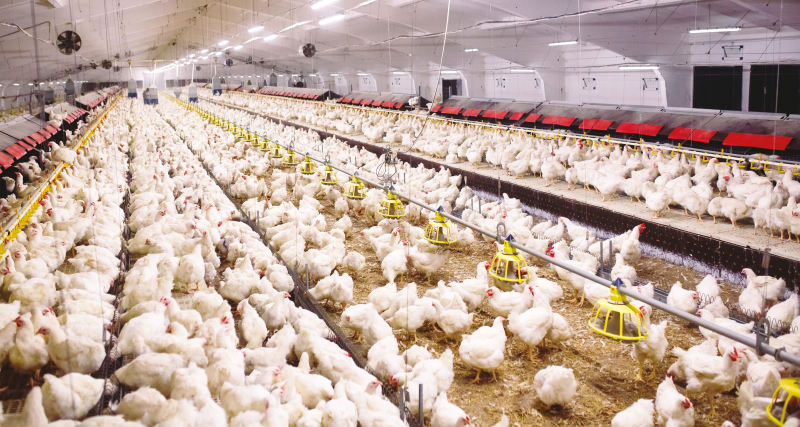
Chicken production in an indoor, cage-free system.

Recognizing animal rights really means accepting that we have a duty not to treat sentient nonhumans as resources. The interesting question is not whether the cow should be able to sue the farmer for cruel treatment, but why the cow is there in the first place.

This proposal may seem radical but in one sense it is not. Most of us already think of animals as having *some* moral value. We may not think of animals as persons, or as having lives that are equal to those of human persons, but we do think that they have a morally significant interest in not suffering. Most of us think that it is morally wrong to impose *unnecessary* suffering on animals.

This belief—that it is wrong to inflict unnecessary suffering on animals—should itself lead us to the conclusion that we should stop virtually *all* of our uses of animals even if we reject the personhood of nonhuman animals. If “necessity” has any meaning in this context, it must mean that we cannot justify inflicting suffering on animals for reasons of pleasure, amusement, or convenience.

There is no necessity for 99% of our animal uses. For example, our numerically most significant use of animals is for food. We kill approximately 70 billion land animals and an estimated one trillion sea animals annually for food. Until recently, it has been accepted in many parts of the world—and especially the West—that eating animals, which accounts for the largest number of animals we use, was necessary for human health. We do not need to consume animals in order to be healthy (Academy of Nutrition and Dietetics, 2019; American Heart Association. 2019; British National Health Service, 2019; Dieticians of Canada, 2019; Kaiser Permanente, 2019; Mayo Clinic, 2019; National Institutes for Health; 2019). Indeed, many mainstream health professionals are claiming that we can be healthier if we adopt a plant-based diet. But whether we will be healthier is not the point, which is that we won’t be less healthy if we do not consume animal products. Eating animals is simply not necessary. The best justification we have for inflicting suffering and death on animals is that we think that they taste good; we derive pleasure from eating them. Eating animals and animal products is a tradition—we have been doing it for a long time (Francione and Charlton, 2013). So what? Patriarchy is a tradition that has existed for a very, very long time. But the fact that something has been happening for a long time says nothing about its moral status. If it is not necessary to consume animal products, then we cannot justify imposing *any* amount of suffering on animals used for food.

And there is no necessity to use animals for clothing, entertainment, or sport. Our *only* use of animals that is even arguably not transparently frivolous involves what is referred to as “vivisection”—using animals in biomedical experiments/contexts to cure serious human illnesses. So even without personhood and the right of animals not to be property, and even if we buy into the anthropocentric fantasy that animals are “inferior” to humans, we would still all be vegans and the only issue we would be discussing would be whether we could justify using animals for experiments, which, under a theory of animal rights, would have to be rejected.

In sum, even if you do not accept the rights position, the position that you probably do accept—that it is wrong to inflict *unnecessary* suffering and death on animals—makes it impossible for you to avoid the conclusion that the use of animals for any purpose that does not involve true compulsion or necessity, including the use of animals for food, clothing, and entertainment, must be ruled out. Any other position relegates animals to the category of things that have no moral value.

## About the Author

**Gary L. Francione** is Board of Governors Distinguished Professor of Law and Katzenbach Scholar of Law and Philosophy at Rutgers University, in New Jersey, United States. He is also Visiting Professor of Philosophy at the University of Lincoln in the U.K. Professor Francione has been teaching animal rights theory and the law for more than 30 years. He has lectured on the topic throughout the United States, Canada, and Europe. He is well known throughout the animal protection movement for his criticism of animal welfare law and the property status of nonhuman animals, and for his theory of animal rights.
